# The study on microstructure and mechanical properties of multi-component composite based on HDPE

**DOI:** 10.1080/15685551.2020.1818956

**Published:** 2020-09-18

**Authors:** Yazhen Wang, Chenglong Wang, Shaobo Dong, Liwu Zu, Tianyu Lan

**Affiliations:** a College of Chemistry and Chemical Engineering, Qiqihar University, Qiqihar, China; b Heilongjiang Provincial Key Laboratory of Polymeric Composite Materials, Qiqihar, China; c College of Chemistry, Chemical Engineering and Resource Utilization, Northeast Forestry University, Harbin, China

**Keywords:** Multi-component, microstructure, crystallization kinetics, mechanical properties

## Abstract

The basalt fiber (BF) and polyamide 6 (PA6) reinforced HDPE composite were prepared; the effects of adding fiber, organic filler, and polar component maleic anhydride (MA) on the microstructural characteristics of composites were investigated. Microstructural characterization evidenced the binary-dispersed phase (PA6/BF) is of a core-shell structure in which the component PA6 encapsulates component BF, and the extent of encapsulates would decline with the MA adding. It is confirmed that the microstructure is related to the interfacial tension of components by the SEM observation and theoretical calculation. The effect of multi-component on the crystallization behavior of composites was investigated. Differential scanning calorimeter (DSC) analyses showed a significant change in the HDPE microstructure. It demonstrated PA6 and BF as a nucleation agent accelerated the crystallization rate under the cooling process. The corresponding crystallization kinetics and activation energy were further analyzed using the Jeziorny method, Avrami–Ozawa method, Kissinger method. The results showed MA markedly changed the crystal growth mechanism of the HDPE matrix to heterogeneous nucleation for acicular and tabular crystal growth during the annealing step. The lowest crystallinity energy and crystallinity were observed for BF/PA6/HDPE composites with 3 wt % MA. Furthermore, a clear improvement of mechanical properties (by 61%) were observed, which mechanism is discussed in detail. The mechanism of toughening is not only one, but the result of a variety of mechanisms together.

## 1. Introduction

High modulus, multiple components, and inexpensive composites have aroused much attention in the related market [1,2]. A simple polymer alone cannot exhibit all the desired characteristics due to the rapid urbanization, highlighting the demand for the multi-component composites. For instance, the importance of improving fuel efficiency has been stressed in the field of automobiles, and there is a gradually increasing demand for lightweight materials. Hence, there is a growing interest in the multi-component composites, particularly in high modulus and high toughness fiber-reinforced polymeric alloy for replacing existing materials [3,4].

At present, the structure and mechanical properties of single-component composites have been studied by many researchers [5]. In general, the properties of composites depend on interfacial bond strength between the fillers and matrix [6]. For example, carbon black and calcium carbonate [7], the large specific surface area, which were added to the polyolefin matrix to improve their interfacial interaction at the molecular level [8]. However, the immiscible multi-component composites systems are more complex [9], and the component of each polymeric phase can form separate phases or slightly encapsulated phases by other dispersed phases [10]. The composites adding fibers or organic fillers will increase the complexity of microstructure, which has an important influence on the properties of the polymer material [10]. Therefore, it is significant to examine the dependency between the morphological structure and mechanical properties of the immiscible multi-component polymer systems.

Lots of studies have contributed to the development of inorganic-reinforced HDPE matrix composites in recent years. The paper of P. Rajeshwari et al. [11] reported the stable crystal structured Aluminum nitride particles were incorporated into HDPE. They found the reason for effective load transfer between HDPE and AIN particles due to homogenous dispersion and superior diffusive bonding on AIN particles. Cagrialp Arslan et al. [12] studied that the effects of three different silane coupling agents on the mechanical properties of the basalt filler (BF) reinforcing polymer. It was observed that the compatibility between filler and matrix was improved, and the reason is that the coupling agent reduced the hydrophilic groups on the BF surface. But these efforts have not been extended to the correlation of the performance between microstructure. Other studies have reported organic filler or fiber reinforcing polymer matrix. Specifically, Lien Zhu and Haoming Wang [13] investigated the mechanical properties and crystallization behavior of HDPE/HDPE-g-MA/PA6 ternary blends. The authors found the dispersed phase is a core-shell structure. The crystallization behaviors are diverse when adding a different composition. Siwon Yu et al. [14] reported that the effects of silanization treatments on the stiffness and toughness of BF/SEBS/PA6,6 hybrid composites. The result shows that the fiber and rubber phases were dispersed separately, thus enhancing both the stiffness and toughness of the composites individually. These efforts, however, the effect of the multi-component on microstructure – property correlation remains unclear. Besides, the correlation between crystallization behaviors and the multi-component composites has not been thoroughly explored in a diversified way.

High-density polyethylene (HDPE) is one of the most common plastics with a wide range of applications. However, its applications were limited due to its low mechanical property [15,16]. Therefore, the aim of this paper is to study the effects of the multi-component fillers on the microstructure and property of HDPE matrix. Using Harkin’s equation to predict the phase of the matrix component and adding component, which is confirmed by the SEM observation and theoretical calculation. Exploring crystallization behaviors of the multi-component composites by differential scanning calorimetry (DSC) analysis, and investigating the stiffening and toughening mechanism of composites.

## 2. Materials and methods

### 2.1. Materials and sample preparation

High-density polyethylene pellets were purchased from Daqing Petrochemical Co. (Daqing, China), density: 0.980 g/cm^3^. Chopped BF supplied by ZhongXing Co. (JiLin, China) was used as the reinforcing material (3 mm). Polyamide 6 (product brand-UBE5034) with a mass density of 1.13 g/cm^3^ was obtained from PoShar Co. (Suzhou, China) Commercial maleic anhydride (MA) was obtained from Aladdin Co, China. Dicumyl peroxide (DCP) was purchased from Aladdin Co, China. HDPE melt grafted MA, denoted as HDPE-MA (The HDPE surface was modified with maleic anhydride in order to provide better compatibility with polar matrices such as PA6). The blends were prepared by melting in a twin-screw extruder. The sample code and composition of each material are listed in Table 1.

**Table 1.** Sample code and composition of the neat polyethylene (PE) and its composites

Code	HDPE	PA6	BF	MA	DCP
Neat HDPE	100	0	0	0	0
HP	60	40	0	0	0
HP_M1_	59	40	0	1	0.1
HP_M2_	58	40	0	2	0.1
HP_M3_	57	40	0	3	0.1
HPB	60	30	10	0	0
HPB_M1_	59	30	10	1	0.1
HPB_M2_	58	30	10	2	0.1
HPB_M3_	57	30	10	3	0.1

### 2.2. Contact angle measurements

Contact angles were measured in a sessile drop mold with a DSA100 (KRÜSS, Hamburg, German). HDPE, HDPE-g-MA, and PA6 samples were compression-molded between clean silicon wafers for 3 min and then cooled to 25°C. The contact angles of each sample were measured using two different liquids (water, ethylene glycol).

### 2.3. Morphology observation

The morphology of all the blends was characterized by scanning electron microscopy (SEM). The samples were fractured in low-temperature brittle fracture immersed in liquid nitrogen then covered with gold (sputtering method) then the fractured samples were observed in the SEM instrument (S-4800, Hitachi, Tokyo, Japan).

### 2.4. Differential scanning calorimetry measurement

To eliminate the thermal and mechanical history and to ensure a nuclei-free melt, the first step preliminary heated the samples to 230°C with a heating rate of 10 °C/min using a NETZSCH DSC-214 (NETZSCH, Germany). Afterward, the samples were cooled to 50 ◦C at different constant cooling rates of 2.5, 5, 10, and 20 °C/min. The exothermic flow curves as a function of temperature were recorded to analyze the non-isothermal crystallization kinetics. The normalized crystallinity (X_C_) of HDPE component was determined by X_C_ = ∆H_m_/(∆H^0^_m_×W_f_), where ∆Hm is the melting enthalpy for the sample at a given cooling rate, ∆H0m denotes the enthalpy of the original polymer crystal (292 J/g for HDPE), and W_f_ is the weight percentage of the PP in the samples.

### 2.5. Tensile testing

The blends were molded at 240 °C with the injection molding machine into dumb-bell shape samples. A universal testing machine ((Nanjing Jieen Te testing instrument CO., LTD, Nanjing, China) was used to test the tensile properties. At least five specimens were tested for each sample to obtain a reliable average and standard deviations for all the mechanical properties. From the stress-strain curves, the following properties (based on averages of samples) were calculated: tensile strength, and elongation at the break where the sample fails.

## 3. Theoretical background

### 3.1. Harkin’s equation

The phase morphology and dispersion have important effects on the properties of composites, and it is necessary to assess the balance between the interfacial properties of the phase. The fundamental concept could be expressed in terms of spreading coefficients, Harkin’s equation could be used to predict the phase of a matrix component and two dispersive components, as follows:

(1) $$\lambda_{BC}=\gamma_{AC}-\gamma_{AB}-\gamma_{BC}$$

(2) $$\lambda_{CB}=\gamma_{AB}-\gamma_{AC}-\gamma_{BC}$$

where A is the matrix, and B and C as the dispersed phases. γ_AB_ is the interfacial tension value between A and B components. When the value of λ_BC_ >0 or λ_BC_ < 0, the component B encapsulates component C and has an adequate interfacial driving force to eliminate the high interfacial energy of matrix component A.

The interfacial tension of each phase can be calculated by the harmonic-mean equation:

(3) $$\left(1+\cos\theta_{H_{2}O}\right)\gamma_{H_{2}O}=4\left(\frac{\gamma_{H_{2}O}^{d}\gamma^{d}}{\gamma_{H_{2}O}^{d}+\gamma^{d}}+\frac{\gamma_{H_{2}O}^{p}\gamma^{p}}{\gamma_{H_{2}O}^{p}+\gamma^{p}}\right)$$

(4) $$\left(1+\cos\theta_{{\left(CH_{2}OH\right)}_{2}}\right)\gamma_{{\left(CH_{2}OH\right)}_{2}}=4\left(\frac{\gamma_{{\left(CH_{2}OH\right)}_{2}}^{d}\gamma^{d}}{\gamma_{{\left(CH_{2}OH\right)}_{2}}^{d}+\gamma^{d}}+\text{\textbackslash{}break}\frac{\gamma_{{\left(CH_{2}OH\right)}_{2}}^{p}\gamma^{p}}{\gamma_{{\left(CH_{2}OH\right)}_{2}}^{p}+\gamma^{p}}\right)$$

(5) $$\gamma_{12}=\gamma_{1}+\gamma_{2}-4\left(\frac{\gamma_{1}^{d}\gamma_{2}^{d}}{\gamma_{1}^{d}+\gamma_{2}^{d}}+\frac{\gamma_{1}^{p}\gamma_{2}^{p}}{\gamma_{1}^{p}+\gamma_{2}^{p}}\right)$$

where γ, γ d and γ p represent the surface tension, the dispersion fractions and the polar fractions, θ is the contact angle of water or ethanediol. γ_12_ represents the interfacial tension between component 1 and component 2, γ_1_ and γ_2_ are the surface tension of two-component in the blends.

### 3.2. Crystallization kinetics

The relative degree of crystallinity X_T_ as a function of crystallization temperature can be calculated using the following equation:

(6) $$X_{T}=\frac{\int_{T_{0}}^{T}\frac{\left(dH\right)}{\left(dT\right)}dT}{\int_{T_{0}}^{T_{f}}\frac{\left(dH\right)}{\left(dT\right)}dT}$$

where T_0_ and T_f_ are the onset and offset temperatures of crystallization, respectively, and dH/dT is the heat flow rate. In non-isothermal crystallization, the arbitrary crystallization temperature T is associated with the crystallization time t through the following equation of the form where β is the cooling rate.

(7) $$t=\left|T-T0\right|/\beta$$

where T_0_ denotes the onset temperature at crystallization time t = 0, T is the temperature at time t, and β is the cooling rate.

The new study derived a new crystallization kinetic equation based on the Avrami Equation (X(t) = 1-exp(-Z_t_t^n^)) [17] and Ozawa Equation (- ln(1-X(t)) =) approaches [18–20]. As following equations:

(8) $$\ln\left[-\ln\left(1-X\left(t\right)\right)\right]=n\ln t+\ln Z$$

(9) $$\log\left[-\ln\left(1-X\left(t\right)\right)\right]=\log k\left(T\right)-m\log\beta$$

where X(t) denotes the relative crystallinity at time t, Z denotes the crystallization rate constant indicating nucleation and growth rate parameters, n is a mechanism constant that explains the nucleation and growing geometry of the crystallites, X(t) is the relative crystallinity at temperature T, k(T) is the cooling crystallization function, m is the Ozawa exponent, which depends on the nucleation mechanism and the crystal growth.

Moreover, considering the influence of the cooling rate β on the crystallization process, the crystallization rate constant (Z) was corrected to obtain the crystallization rate (Z_C_) as a function of the cooling rate (β) according to the following equation:

(10) $$\ln Z_{C}=\left(\ln Z\right)/\beta$$

However, the Ozawa equation does not apply to describe the non-isothermal crystallization kinetics of some polymer systems. Therefore, by combing the Avrami and Ozawa equations, the equation related to the cooling crystallization rate β and time t [17,21]. Can further be rewritten as:

(11) $$\ln\beta =\ln F\left(T\right)-\alpha \ln t$$

where F(T) = [k(T)/K]1/m refers to the value of cooling or heating rate at the unit crystallization time under a given certain relative degree of crystallinity, and α denotes the ratio of the Avrami exponent n to the Ozawa exponent m.

The crystallization activation energy was calculated by the Kissinger equation, and the equation is expressed as follows [22]:

(12) $$\frac{d\left[\ln(\beta /T_{p}^{0}\right)]}{d\left(1/T_{P}\right)}=\frac{-\Delta E}{R}$$

where T_p_ denotes the crystallization peak, ∆E is the activation energy, which characterizes the transport process of macromolecular segments to the surface of crystal growth, R is the universal gas constant.

## 4. Results and discussion

### 4.1. Morphology prediction based on the phase behavior of the multi-component composites

Table 2 summarizes the surface tensions of each component by Equations (3) and (4), and the results were measure based on the contact angle. Equation (5) was used to calculate the interfacial tensions, which are listed in Table 3. As can be shown from Tables 2 and 3, the surface tension of PA6 is 50, of which value is between HDPE and BF. The interfacial tension of HDPE/BF is 28.5, which is much higher than that of PA6/BF (4.2) and HDPE/PA6 (13.0). In the HPB blends, we can speculate that PA6 will encapsulate BF forming core-shell morphology due to the free energy of multicomponent polymer system is usual minimum.

**Table 2.** Calculated of contact angle and surface tension data of the blends

Sample	Contact	Angle	Surface Tension		(mN/m)
	Water	Ethanediol	Total(γ)	Dispersion Component(γ^d^)	Polar Component(γ^p^)
HDPE	93.37	69.16	24.16	14.66	9.94
PA6	51.00	29.39	50.00	15.82	34.23
HDPE-MA_1_	87.09	61.63	28.10	15.78	12.33
HDPE-MA_2_	80.17	58.76	30.78	13.29	17.49
HDPE-MA_3_	78.27	57.91	31.8	12.82	18.92
BFs	–	–	72.50 [16]	21.50	51.00

**Table 3.** Interfacial tension of the blends

Polymer Pairs	Interfacial Tension (mN/m)
HDPE/PA6	13.0
HDPE-MA_1_/PA6	10.3
HDPE-MA_2_/PA6	5.6
HDPE-MA_3_/PA6	4.8
HDPE/BF	28.5
HDPE-MA_1_/BF	24.5
HDPE-MA_2_/BF	18.3
HDPE-MA_3_/BF	16.9
PA6/BF	4.2

Luzinov et al. [23] used Equation (1) and (2) to predict phase morphology. In the case of HPB ternary system, the spreading coefficients for λ _BF/PA6_ and λ _PA6/BF_ were −19.7 and 11.3, the form where the dispersive phase component PA6 exists partially at the interface between another dispersive phase component BF and matrix component HDPE would be taken. Furthermore, when the maleic anhydride was used to treat HDPE, the spreading coefficients of HPB_M1_ blends were −18.2 for λ _BF/PA6_ and 10 for λ _PA6/BF_, which was lower than HPB blends, this is due to the chemical reaction between the anhydride group of the MA and the molecular chain of HDPE, which increases the polar component of the material. The interfacial tension between HDPE-MA_1_ and PA6 decreases so that the PA6 has a sufficient thermodynamic driving force to remain in the interphase between the BF and HDPE-MA_1_. In this case, it contributes to improving the compatibility between PA6 and the matrix. The HPB_M1_ composites appear to have a core-shell morphology based on Equation (1) and (2) calculated.

In the case of HPB_M2_ blends, similarly, it can be found that the value of the spreading coefficient for λ _BF/PA6_ and λ _PA6/BF_ decreases. This is because of the decline of interfacial tension for HDPE-MA_2_/PA6, and leads to PA6 phase that may shift to the matrix. The HPB_M2_ composites appear to have a core-shell morphology. In contrast, For the HPB_M3_ blends, the interfacial tension value for HDPE-MA_3_/PA6 and PA6/BF was 4.8 and 4.2. Therefore, it was predicted that the BFs and PA6 would have separate dispersed phase structures, with dispersive phase components distributed individually throughout the matrix component HDPE-MA_3_. The reason is similarly interfacial tension, and thus PA6 phase exists at the interface between BF phase and matrix HDPE-MA_3_. Another reason was the higher interfacial tension of HDPE-MA_3_/BF (16.9), which has a sufficiently high tension to make the component BF removed the matrix component HDPE-MA_3_. Therefore, we speculate that the purpose of dispersing the enhanced phase can be achieved by controlling the polarity of the material, which is conducive to achieving a balance between properties.

### 4.2. Morphology observation of the multi-component composites

**Figure 1.**
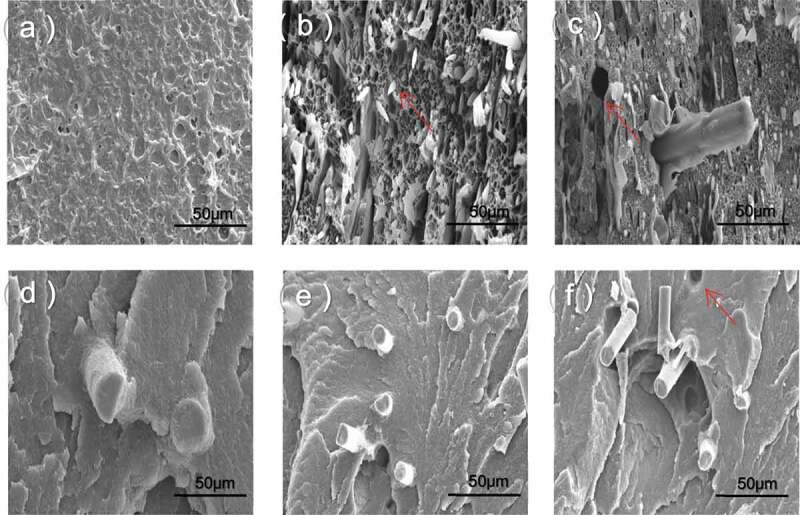
SEM images of the fractured surfaces of the composites after impact test. (a) HDPE; (b) PA6/HDPE; (c) BF/PA6/HDPE; (d) BF/PA6/HDPE-MA_1_; (e) BF/PA6/HDPE-MA_2_; (f) BF/PA6/HDPE-MA_3._

Figure 1 shows the fracture surfaces of composites after the impact test. The SEM image (Figure 1(a)) of pure HDPE shows a relatively smooth surface and comparatively complete. From Figure 1(b), a clear number of pull-out holes between the dispersed phase and the matrix are observed, indicating poor interfacial adhesion between HDPE and PA6, we can speculate that the blends do not have a sufficient chemical affinity to combine into a completely miscible system. In addition, HDPE and PA6 are crystallization polymers, and the crystallization process will lead to volume shrinkage. However, this poor compatibility was improved significantly by the MA treatment (see Figure 1(d-e)). The BF/PA6/HDPE composites shown in Figure 1(c) indicate that small PA6 microdomains were predominantly distributed through the HDPE matrix and move towards BF reinforcement, taking into account the above morphological prediction and SEM observe, it could be convinced that PA6 encapsulate BF, forming a core-shell structure. When used the reactive MA modifier, the interfacial properties between PA6 and matrix, the BF and PA6 underwent significant changes (see Figure 1(d)). It can be observed that BF is surrounded by the matrix. The interphase boundaries between the three phases became indistinct, and they became a highly miscible system. This is because the reaction between the anhydride group of HDPE-MA_1_ and the amino end group of partial PA6 would be taken. This resulted in a phase transformation, indicating the link between component HDPE-MA1 and component PA6 is not only the intermolecular force and the molecular chain entanglement but also covalent bond. With the amount of maleic anhydride, the polymer matrix around BF decreases significantly (see Figure 1(e)), The reason for this phenomenon is that more maleic anhydride groups increased the polar of the matrix, leading to the chemical affinity between PA6 and HDPE-MA_2_ improves. Furthermore, the component PA6 will be closer to component HDPE-MA_2_ due to the interface tension of component PA6, and component BF is unchanged. This weakened the interaction between the PA6 and BF.

However, the morphology of the HPB_M3_ composite was different. As shown in Figure 1(f), it was observed that the BF had a separate dispersed phase structure. The surface of BF was smooth and did not attach the other dispersed phase. These results are consistent with previous predictions of the morphologies. That is, a higher amount of maleic anhydride affects the polarity and chemical affinity of each component, thus preventing encapsulation by the dispersed phase. The results of scanning electron microscopy (SEM) imaging clearly showed that the predictions of the morphologies based on the interfacial energies were reasonable. Consequently, it is feasible to control the dispersion of the enhanced phase by adjusting the polarity of the material.

### 4.3. Crystallization behavior and kinetic analysis

#### 4.3.1. Isothermal crystallization behavior

**Figure 2.**
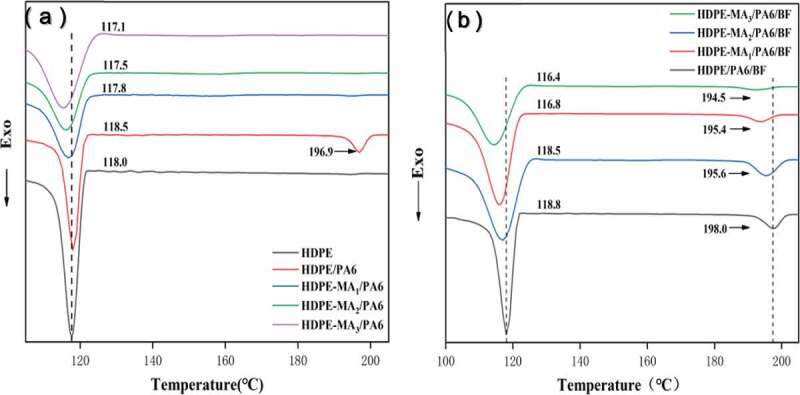
The DSC cooling curve of composites. (a) binary blends. (b) multi-component blends

As shown in Figure 2(a), the neat HDPE have crystallization peak at 118°C, while the crystallization peak of the blends HP and HPMX have distinct change. It can be observed two distinct crystallization peaks for HDPE/PA6 blend, with the crystallization at 196.9°C corresponding to PA6 crystallization and crystallization at 118.5°C corresponding to HDPE crystallization. Compared with the neat HDPE, the crystallization temperature of HDPE in the blend increases. The reason is that PA6 crystallize at high temperatures which could act as nucleating agents for HDPE crystallization at low temperatures and accelerate the crystallization process. On the other hand, the PA6 crystallization peak has disappeared in the HP_M1_ blends and appeared a compatible crystalline peak. The result indicated the value of crystallization peak lower, and other samples showed the same tendency. This result probably suggested that the transport of HDPE molecular chain was impeded at the crystal surface due to the chemical reaction between the anhydride group of MA and the amino-terminal group of the PA6, which decreased the overall flexibility and regularity of the molecular chain. Furthermore, the polarity of the matrix would be higher with the amount of MA increases, which makes polar groups of the molecular chain entangled with each other, which could result in less entropy and thereby decreases the enthalpy change and reduced crystallization ability.

As shown in Figure 2(b), the blend HPB has two crystallization peaks at 118.8°C and 198.0°C corresponding to HDPE and PA6. Compared with the binary blend HP, the increase in crystallization temperature is owing to the BF could act as a heterogeneous nucleation agent for the crystallization of HDPE and PA6 in the blend. As it is seen from the HPBM1 blend that the split-new crystal peak appears at 195.6°C compared with the HDPE-M1/PA6 sample (see Figure 2(a)). This result clearly indicated that PA6 preferentially adheres to the BF surface for crystallization, due to the interfacial tension between PA6 and BF lower compared with that between PA6 and HDPE-MA_1_(see Table 2). The same situation for HPB_M2_ and HPB_M3_ blend. In the HPB_M1_ blend system, the crystallization temperature distinctly decreases compared with HPB blend. This phenomenon can be due to the addition of MA makes the chemical affinity between HDPE and PA6 rising, which led to the molecular chain movement was impeded [24,25].

#### 4.3.2. Non-isothermal crystallization behavior

**Figure 3.**
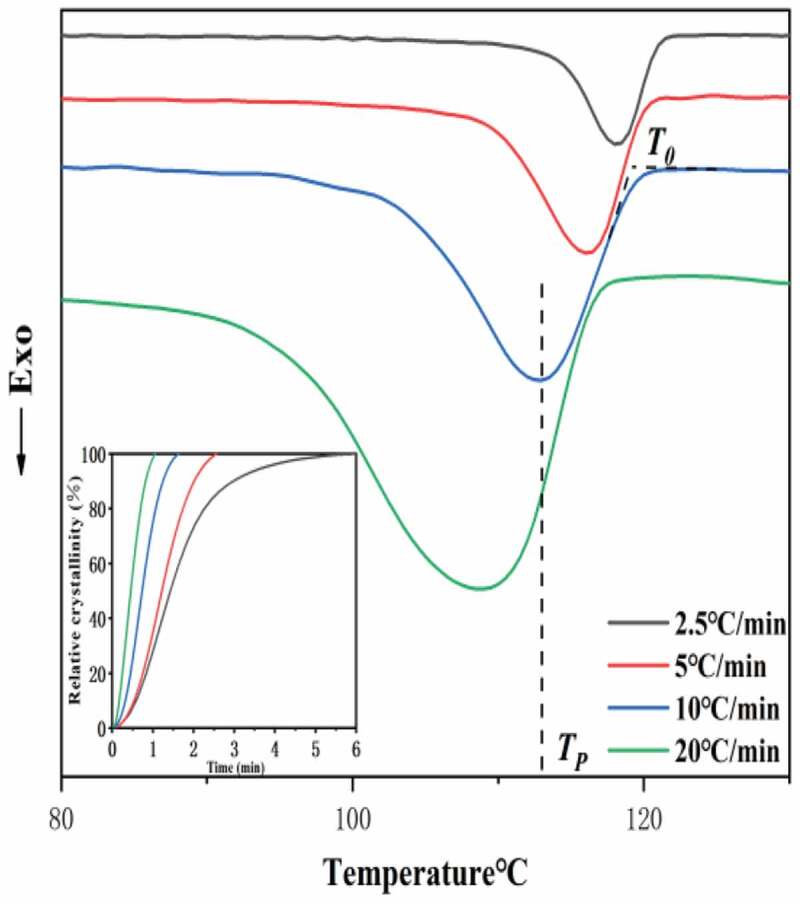
Non-isothermal crystallization behaviors of the neat HDPE. Differential scanning calorimetry (DSC) thermograms. (the inset is Relative crystallinity as a function of time.)

To further validate the crystallization process, Figure 3 shows the DSC cooling curves of the non-isothermal crystallization of neat HDPE for a range of cooling rates from 2.5 to 20°C/min. It can be seen that the range of crystallization curves broadened, the onset temperature (T_0_) and the crystallization peak (T_P_) shift to lower temperatures with an increasing cooling rate. This result is attributed to the fact that a lag phenomenon off the molecular chain movement in the process of increasing the cooling rate. In contrast, sufficient time to form crystal nuclei for random molecular chains at the lower cooling rate and crystallization occurred at higher temperature [24,26]. The relative crystallinity (X_C_) as a function of the crystallization time (t) for the neat HDPE at various cooling rates are presented in the inset. It can be shown that the curves showed sigmoidal growth and the same situation for all the samples at different cooling rates. This phenomenon proved the lag effect of the cooling rate on the crystallization. During the beginning stage of the curve [27], it shows that the crystallization rate slowly increased, indicating the crystal nucleus is not easy to form in the early stage due to molecular thermal motion. The crystallization rate decreased in the later stage of the curve owing to the impingement of spherulites [17,28]. Furthermore, at the higher cooling rate, the curve slope was increased compared to the lower cooling rate, this phenomenon indicating the time of crystallization completion increased with decreasing cooling rates.

**Figure 4.**
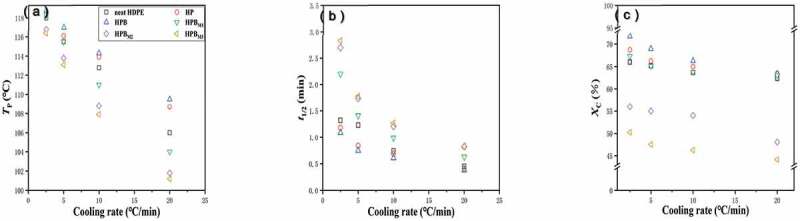
Non-isothermal crystallization parameters as a function of the cooling rate for the neat HDPE and composites with various maleic anhydride (MA) concentrations. (a) The crystallization peak (T_p_); (b) The half-time (t_1/2_); (c) Crystallinity (Xc)

As shown in Figure 4(a), at the given cooling rate, the T_P_ values of MA-containing samples were lower than those of neat HDPE, especially for the HP and HPB. The results indicated that the MA could delay the crystallization process. On the other hand, as shown in Figure 4(b), the t_1/2_ (the period corresponding to an Xc of 50%) value of samples was gradually decreased with an increase in the cooling rates. Furthermore, it can be shown that the t_1/2_ value of various MA-containing samples was higher than that of others, and the effect of MA is obviously seen in the case of a lower cooling rate. At the cooling rate of 2.5 °C/min, the t_1/2_ value decreased to 1.18 min when PA6 was added to the HDPE matrix, and this value further decreased to 1.09 for the HPB ternary composites. In contrast to neat HDPE, it can be speculated PA6 and BF could act as a heterogeneous nucleation agent to accelerate the crystallization rate. However, a clearly increased t_1/2_ value was observed with MA content added. For instance, the t_1/2_ value raised to 2.2 min as MA content increases up to 1 wt %, and this value gradually improved to 2.8 min for 3 wt %. This result may stem from the fact that the rearrangement of molecular chains is hindered, owing to MA makes polar groups of the molecular chain increased, and resulting in a reduced crystallization of the HDPE matrix.

Figure 4(c) reveals that the crystallinity (Xc) value of the neat HDPE decreased from 66% (2.5°C/min) to 62.3% (20°C/min) with increasing cooling rates, and the same situation was shown to the other samples. When PA6 and BF were incorporated in the HDPE matrix, the X_C_ value increased by 6–10%, suggesting that the PA6 and BF could clearly increase the crystallinity of the HDPE matrix. By contrast, the X_C_ values of the MA-containing samples were gradually lower than those of the neat HDPE at various cooling rate. This result likely indicated that the chain length of HDPE was decreased owing to the high polar groups of MA for matrix; therefore, the mobility of HDPE molecular chains was impeded at the crystal surface, and they could not rearrange themselves into ordered form. It is consistent with the findings of the previous researcher [23].

#### 4.3.3. Kinetic analysis with the Jeziorny approach

**Figure 5.**
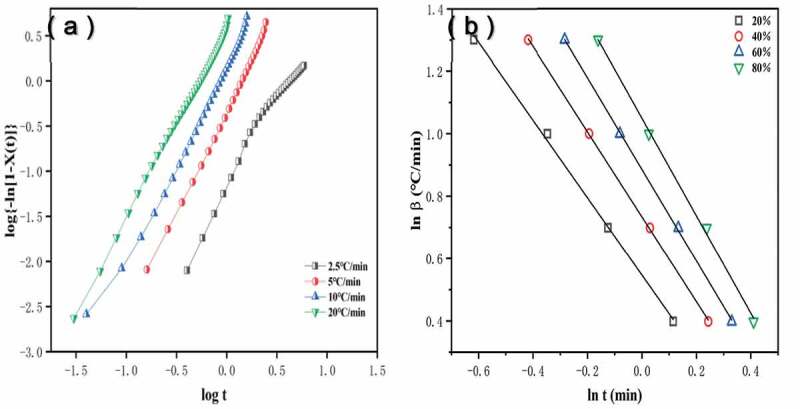
The non-isothermal crystallization kinetics of the neat HDPE. (a) Jeziorny plots of ln[–ln(1–X)] versus ln t; (b) Avrami–Ozawa plots of ln β versus ln t

The nucleation mechanisms were further analyzed based on the Avrima approach modified by Jeziorny in the initial stage of non-isothermal crystallization. At the given cooling rate, the curves of ln[-ln(1-X(t))] versus ln t can be used to calculate the values for n and Z from the slope of the linear portion and intercept based on the formula (8). Figure 5(a) shows linear fits of the plots describing the primary stage of crystallization and the square of the correlation coefficient (R^2^) value which was greater than 0.93 (Table 4), and the curves of log[-ln(1-X(t))] versus log t not totally linear relation. The reason is the initial crystal nucleus form at this stage and crystallize slowly in the crystallization induction period. On the other hand, the partial fitting lines of the neat HDPE were almost parallel and the same tendency for other samples (not shown). It was indicating that the nucleation mechanism and geometry of crystal growth at different cooling rates were similar. In addition, the n values ranged from 1.90 to 2.85 for the neat HDPE, indicating that its non-isothermal crystallization corresponded to two-dimensional tabular crystal growth. The n values of HP and HPB samples were nearly 1.6, suggesting that its crystal growth corresponded to acicular, and the same tendency for the samples HPB_M1_ and HPB_M2_. At the cooling rate of 2.5 °C/min, the n values of HPB are slanted big, which probably means the occurrence of the secondary crystallization process. However, the following n values of HPB_M3_ were in the range of 1.96–2.04, indicating that its crystallization mechanisms were acicular and tabular crystal growth in precedence to heterogeneous nucleation. These results indicated that the addition of PA6, BF and MA markedly changed the nucleation mechanism of the HDPE matrix under the crystallization process.

As presented in Table 4, the Z_C_ values for various samples significantly increased with growing cooling rates up to 5–10 °C/min, and this result has shown that the highest cold crystallization rate occurs at 5–10 °C/min. Furthermore, it shows that the HP and HPB nearly exhibited the highest Z_C_ values among all samples at given different cooling rates. It was indicating the fact that PA6 and BF could be acted as nuclei to improve the crystallization of the HDPE matrix. In contrast, the Z_C_ values for MA-containing samples gradually reduced with the increasing MA concentration. This result is probably attributed to the fact that MA makes polar groups of the partial HDPE matrix increased, the mobility of molecular chains was impeded, and the chemical reaction between the anhydride group and the amino-terminal group of the PA6, which hinders the rearrangement of chains.

#### 4.3.4. Kinetic analysis with the Avrami–Ozawa approach

To explore the crystallization behavior, a new kinetic equation by combining the Avrami equation with Ozawa equation were, which were analyzed the overall non-isothermal crystallization. The F(T) and α can obtain from the linear slope and intercept according to Equation (11). At a given degree of crystallization, the curves of lnα and lnt for neat HDPE was presented in Figure 5(b), in addition, the corresponding data for each sample are listed in Table 5 . The R^2^ value was greater than 0.97, indicating the good linearity verifies the successful application of this combined method in this case. The value for F(T) steadily increased with the increase of relative crystallinity X_T_, and the values of α were nearly invariable. Thence, a given higher cooling rate at the unit crystallization time can be used to obtain the higher crystallinity. Additionally, the F(T) values of HP and HPB were lower than those of the neat HDPE at the given relative crystallinity. This is indicative of a faster crystallization process. Among the MA-containing samples, the F(T) values gradually rise with the increasing MA concentration and it is consistent with the results of the Jeziorny approach given above. These results indicated that a slower cooling rate can achieve a higher degree of crystallinity for PA6 and BF filling with HDPE due to the effect of heterogeneous nucleation, and it is difficult to obtain a higher degree of crystallinity for HPB_M3_ at the lower cooling rate.

**Table 4.** The parameters calculated at various cooling rates based on the Jeziorny model

code	Cooling Rate (°C/min)	Z	Z_C_	n	R^2^
Neat PE	2.5	0.05 ± 0.01	0.30 ± 0.02	2.85 ± 0.32	0.9379
	5	0.44 ± 0.01	0.85 ± 0.01	2.23 ± 0.02	0.9991
	10	1.14 ± 0.07	1.01 ± 0.01	2.01 ± 0.04	0.9929
	20	3.03 ± 0.16	1.06 ± 0.02	1.90 ± 0.03	0.9908
HP	2.5	0.52 ± 0.02	0.77 ± 0.01	1.84 ± 0.05	0.9975
	5	0.96 ± 0.05	0.99 ± 0.01	1.76 ± 0.05	0.9940
	10	1.33 ± 0.03	1.03 ± 0.01	1.66 ± 0.04	0.9947
	20	2.58 ± 0.27	1.05 ± 0.01	1.50 ± 0.06	0.9819
HPB	2.5	0.24 ± 0.01	0.56 ± 0.01	2.49 ± 0.05	0.9983
	5	0.71 ± 0.05	0.93 ± 0.01	1.54 ± 0.01	0.9995
	10	1.34 ± 0.11	1.03 ± 0.01	1.28 ± 0.06	0.9802
	20	0.84 ± 0.05	0.99 ± 0.01	1.16 ± 0.06	0.9682
HPB_M1_	2.5	0.14 ± 0.01	0.46 ± 0.01	1.95 ± 0.02	0.9989
	5	0.37 ± 0.01	0.82 ± 0.02	1.88 ± 0.02	0.9980
	10	0.72 ± 0.02	0.97 ± 0.01	1.84 ± 0.03	0.9957
	20	1.67 ± 0.08	1.03 ± 0.01	1.91 ± 0.04	0.9934
HPB_M2_	2.5	0.10 ± 0.01	0.40 ± 0.01	1.88 ± 0.04	0.9940
	5	0.24 ± 0.01	0.75 ± 0.02	1.95 ± 0.02	0.9979
	10	0.47 ± 0.01	0.93 ± 0.01	1.92 ± 0.02	0.9967
	20	1.00 ± 0.03	1.00 ± 0.01	1.96 ± 0.03	0.9948
HPB_M3_	2.5	0.10 ± 0.01	0.40 ± 0.01	1.98 ± 0.02	0.9992
	5	0.22 ± 0.03	0.74 ± 0.02	2.04 ± 0.01	0.9990
	10	0.42 ± 0.01	0.92 ± 0.01	2.01 ± 0.03	0.9961
	20	0.81 ± 0.02	0.99 ± 0.01	1.96 ± 0.02	0.9964

**Table 5.** The parameters calculated at various relative crystallinities (X) based on the Avrami–Ozawa model

code	X%	F (T)	α	R^2^
Neat PE	20	3.52 ± 0.10	1.23 ± 0.03	0.9973
	40	5.40 ± 0.03	1.35 ± 0.01	0.9998
	60	7.67 ± 0.05	1.46 ± 0.01	0.9997
	80	10.96 ± 0.25	1.56 ± 0.04	0.9985
HP	20	1.37 ± 0.62	1.85 ± 0.05	0.9983
	40	2.80 ± 0.39	1.96 ± 0.23	0.9742
	60	5.16 ± 0.60	1.99 ± 0.28	0.9604
	80	11.75 ± 2.34	2.58 ± 0.52	0.9605
HPB	20	2.30 ± 0.31	1.12 ± 0.16	0.9796
	40	3.77 ± 0.10	1.41 ± 0.01	0.9998
	60	6.10 ± 0.28	1.58 ± 0.12	0.9931
	80	10.41 ± 1.82	1.64 ± 0.32	0.9626
HPB_M1_	20	3.62 ± 0.15	1.70 ± 0.07	0.9960
	40	7.29 ± 0.21	1.68 ± 0.06	0.9969
	60	11.60 ± 0.34	1.63 ± 0.05	0.9978
	80	17.63 ± 0.61	1.58 ± 0.04	0.9973
HPB_M2_	20	5.53 ± 0.21	1.70 ± 0.08	0.9922
	40	10.89 ± 0.42	1.69 ± 0.07	0.9950
	60	17.46 ± 0.92	1.69 ± 0.08	0.9958
	80	27.18 ± 2.15	1.68 ± 0.08	0.9949
HPB_M3_	20	5.59 ± 0.18	1.82 ± 0.73	0.9967
	40	11.40 ± 0.36	1.79 ± 0.06	0.9974
	60	18.31 ± 0.75	1.75 ± 0.06	0.9972
	80	28.85 ± 1.32	1.76 ± 0.05	0.9974

#### 4.3.5. Activation energy of non-isothermal crystallization by the kissinger method

**Figure 6.**
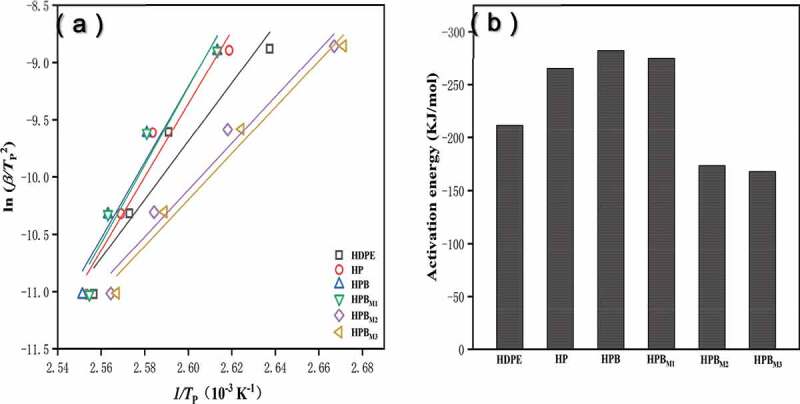
The non-isothermal crystallization kinetics of the neat HDPE and HPB_S_ using the Kissinger method. (a) Kissinger plots of ln (β/T_p_^2^) versus 1/T_p_^2^; (b) the crystallization activation energy

It is essential for polymer systems to calculate the crystallization energy. The crystallization of a polymer matrix in composites is influenced by the following two factors: the static factor, which is related to the free barrier energy of nucleation, and the dynamic factor, which corresponds to the activation energy for the transport of the macromolecular segments to the surface of crystal growth. The Kissinger kinetic model is used to estimate polymers crystallization activation energy by many researchers [29]. In non-isothermal systems, was calculated in this study, using the Kissinger equation (Equation (12)). Figure 6(a) reveals straight-lines of the Kissinger in a ln (β/T_p_^2^) versus 1/T_p_^2^ plot for the neat HDPE and HPBS, enabling the good linear regression for all samples to obtain the ∆E value. Therefore, as shown in Figure 6(b), the ∆E values were −265.51 kJ/mol for HP and −282.26 kJ/mol for HPB, which are lower than that of neat HDPE (−211.61) owing to the nucleating effect of PA6 and BF. However, the ∆E values were further increased to −173.35 kJ/mol and −167.74 kJ/mol when 2 wt %-3 wt % MA was added to the blends. These results indicated that PA6 and BF could promote the crystal growth of the polymer matrix, in contrast, excessive MA would hinder the transport of the polymer chain segments to the surface of crystal growth. It is consistent with the results described above.

### 4.4. Mechanical performance analysis

**Figure 7.**
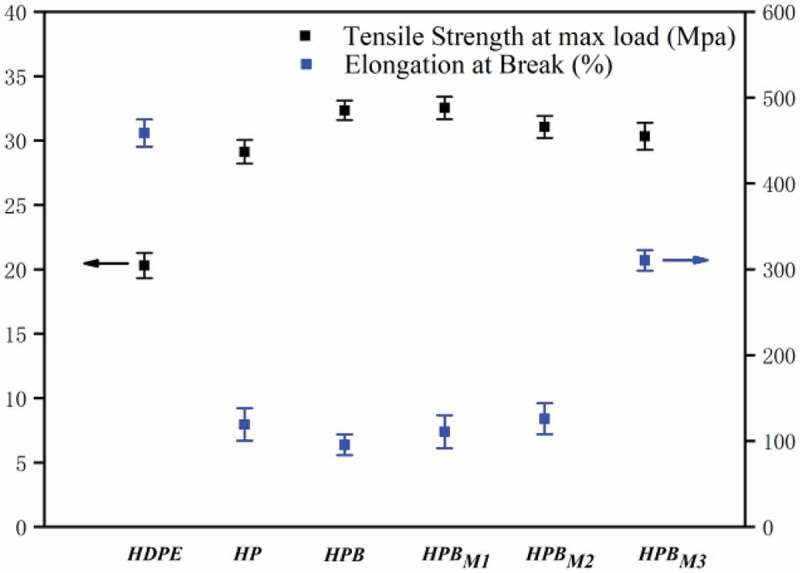
Stress-strain curves of composites

Figure 7 reveals the stress–strain curve of the composites. As expected, the PA6 and BF had a significant effect on the mechanical performance of the composites. In contrast to neat HDPE, the tensile strength of HP and HPB composites has been sharply increased. It is a contributing factor that BF and PA6 are the reinforcing material with more stiffness and strength than neat HDPE. Furthermore, another reason is the addition of BF and PA6 increases the degree of the composite of crystallinity based on the conclusion above. The MA-containing composites degraded their tensile strength with the increasing MA concentration, but the strain at break gradually increased. This may be because of the chemical reaction between the anhydride group and PA6, thereby reducing theability of the rearrangement of chains and increased the amorphous polymer area.

**Figure 8.**
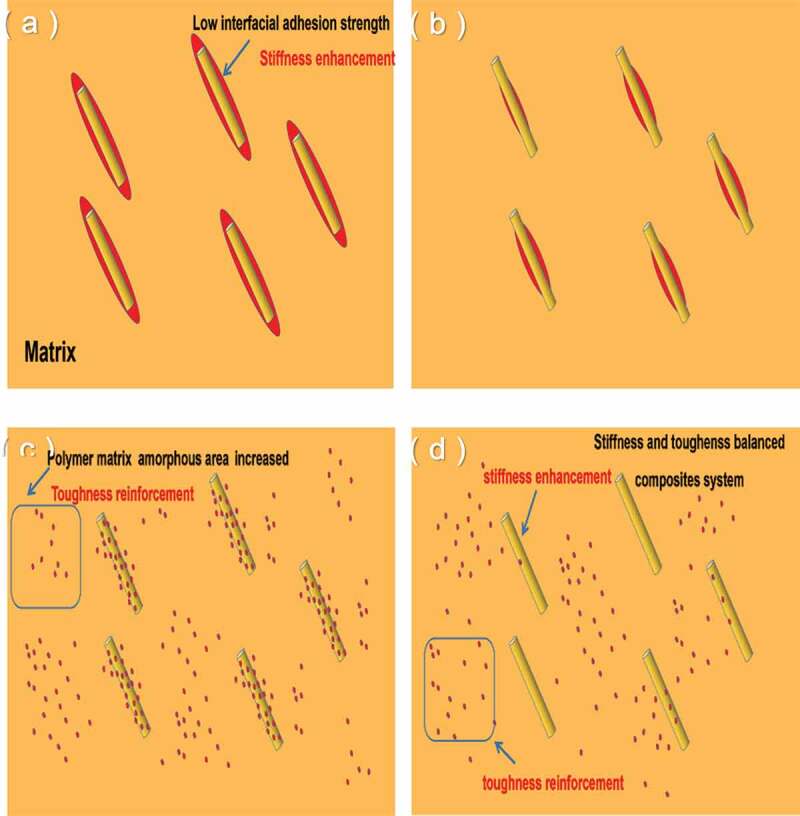
Schematic representation of stiffening and toughening mechanism. (a) HPB; (b) HPB_M1_; (c) HPB_M2_; (d) HPB_M3._

On the other hand, mechanical performance can be affected by the polar component of polymers. As shown in Figure 8(a), it is a core-shell morphology that the component PA6 encapsulates component BF based on the above conclusions for HPB composites, and there is poor interface adhesion strength between PA6 and HDPE matrix. Thus, the toughness of HPB composites sharply decline. As shown in Figure 8(b-d), the interface adhesion strength was improved between PA6 and HDPE matrix with the increasing MA concentration, leading to the ability of the rearrangement of chains decreased. Because of this, the amorphous area of polymer matrix gradually increases, and the toughness of composites will be marked rise. The stiffness and toughness balanced composite system will appear when adding the appropriate MA concentration.

## 5. Conclusions

Through the system design of the multi-component fillers, the composites with simultaneously improved stiffness and toughness were successfully fabricated. The Harkin’s Equation is used to calculate the phase of the matrix component and adding component, and the HPB composites is of a core-shell structure. The phase morphology has changed significantly after adding the polar component MA. It is confirmed for reasonableness of prediction by the SEM observation. According to the Jeziorny method the addition of PA6 and BF significantly changed the nucleation mechanism of the HDPE matrix from acicular crystal growth to acicular and tabular crystal growth under the cooling process. Additionally, in the multi-component system, the Avrami–Ozawa method indicated a slower cooling rate could achieve a higher degree of crystallinity for PA6 and BF filling with HDPE due to the effect of heterogeneous nucleation, and it is difficult to obtain a higher degree of crystallinity for HPB_M3_ at the lower cooling rate. Furthermore, the Kissinger method indicated that the crystallization activation energy (∆E) decreased when BF, PA6 and MA were added to the HDPE matrix, and the HPB_M3_ exhibited the highest ∆E value among all samples. Our results clarified the relationship between the microstructure and the mechanical properties of the multi-component composites. The method of adding multi-component-dispersed phase increased the interfacial bonding strength and expanded the amorphous region. We expect that our findings will contribute to the development of high-performance engineering materials with balanced engineering properties. This will increase the range of engineering-related applications of these materials within fields such as automobiles, machinery, and aerospace.
